# Modeling Lysosomal Storage Diseases in the Zebrafish

**DOI:** 10.3389/fmolb.2020.00082

**Published:** 2020-05-06

**Authors:** T. Zhang, R. T. Peterson

**Affiliations:** Department of Pharmacology and Toxicology, College of Pharmacy, University of Utah, Salt Lake City, UT, United States

**Keywords:** Lysosomal storage disease, zebrafish, genetics, metabolism, chemical screening, CRISPR-Cas9

## Abstract

Lysosomal storage diseases (LSDs) are a family of 70 metabolic disorders characterized by mutations in lysosomal proteins that lead to storage material accumulation, multiple-organ pathologies that often involve neurodegeneration, and early mortality in a significant number of patients. Along with the necessity for more effective therapies, there exists an unmet need for further understanding of disease etiology, which could uncover novel pathways and drug targets. Over the past few decades, the growth in knowledge of disease-associated pathways has been facilitated by studies in model organisms, as advancements in mutagenesis techniques markedly improved the efficiency of model generation in mammalian and non-mammalian systems. In this review we highlight non-mammalian models of LSDs, focusing specifically on the zebrafish, a vertebrate model organism that shares remarkable genetic and metabolic similarities with mammals while also conferring unique advantages such as optical transparency and amenability toward high-throughput applications. We examine published zebrafish LSD models and their reported phenotypes, address organism-specific advantages and limitations, and discuss recent technological innovations that could provide potential solutions.

## Introduction

Lysosomal storage diseases (LSDs) are a family of 70 metabolic disorders caused by mutations in lysosomal proteins that lead to lysosomal dysfunction (Platt et al., 2018). Loss-of-function mutations in lysosomal proteins disrupt lysosomal catabolism, triggering progressive accumulation of substrate materials, and multiple-organ pathologies (Platt et al., 2018). LSD symptoms fall along a spectrum, with early-onset forms typically associated with more severe outcomes: LSDs that arise in infancy often involve neurodegeneration and lead to death within the first few years of life in a significant number of patients (Platt et al., 2018). While some enzyme replacement therapies and small-molecule pharmaceuticals have been approved as LSD therapeutics, the majority of the currently known LSDs lack treatment options (Beck, 2018).

As part of an effort to more thoroughly evaluate the mechanisms of LSD progression, which could yield novel pathways as potential drug targets, genetic models of LSDs now encompass a wide range of biological systems. While mammalian (Xu et al., 2016; Gurda and Vite, 2019) and cell-based (Borger et al., 2017; Frati et al., 2018) models have contributed significantly to the understanding of LSD etiology, a survey of the existing literature also highlights a rise in LSD models in the zebrafish, a vertebrate organism that shares remarkable similarities with mammalian systems while also conferring unique advantages such as optical transparency and amenability toward high-throughput screening (Rennekamp and Peterson, 2015; Meyers, 2018). Importantly, recent advancements in gene editing methods such as TALENs (Sander et al., 2011; Hwang et al., 2014), and CRISPR-Cas9 (Hwang et al., 2013a, b; Gagnon et al., 2014) have significantly improved the specificity and speed of targeted model generation, and will likely lead to a further upsurge in the availability of zebrafish LSD models.

In this review, we highlight the zebrafish model organism as an emerging candidate for LSD model generation. In surveying the literature, we identified 60 zebrafish LSD models with published phenotypic data, many of which recapitulate known human pathologies. The reported models include transient knockdowns and stable mutants, and belong to eight categories including sphingolipidoses, mucolipidoses (MLs), neuronal ceroid lipofuscinoses, integral membrane protein disorders, glycogen storage diseases (GSD), glycoproteinoses, mucopolysaccharidoses (MPS), and lysosome-related organelle disorders (Platt et al., 2018). In the following sections of this review we examine these models in detail. We also provide a brief overview of the zebrafish model system in which we discuss comparative anatomy, genetics, and the current toolkit for phenotypic characterization. We discuss organism-specific advantages and shortcomings, and present innovations that could address current limitations.

## The Zebrafish Model Organism

Since the initial implementation of zebrafish in developmental biology and genetics research in the 1980s (Streisinger et al., 1981), the subsequent decades have witnessed a dramatic rise in zebrafish-focused projects, from four zebrafish-related publications in 1990 to 3829 in 2019 (Pubmed, 1996, search term: zebrafish, publication date range: 1/1/19–12/31/19, and date of query: 1/30/20; Pubmed, 1996). In the following subsections we present a brief overview of the zebrafish system in the context of disease modeling, focusing on comparative anatomy, genetics, and toolkit for model characterization.

### Comparative Anatomy

The zebrafish (*Danio rerio*) is a small, freshwater fish native to South Asia, and is one of the more than 20 species belonging to the genus *Danio* (McCluskey and Braasch, 2020). Zebrafish are teleosts, a diverse infraclass that includes approximately 30000 named species (Witten et al., 2017). Anatomically, major zebrafish organs include the eyes, brain, gills, teeth, otolith, heart, thyroid gland, thymus, spleen, kidney, interregnal, and chromaffin cells (counterparts to the mammalian adrenal cortex and adrenal medulla, respectively), corpuscle of stannous, ultimobranchial gland, pancreas, liver and gallbladder, white adipose tissue, intestine, swim bladder, and organs of the reproductive system (Menke et al., 2011). Zebrafish organogenesis occurs rapidly: major organs become fully functional after the first few days of life, with development continuing through the juvenile stage.

A summary of the notable similarities and differences between major zebrafish organs and human counterparts is shown in Table 1. A significant number of tissue and cell types analogous to those found in humans also exist in the zebrafish, while other key aspects of mammalian anatomy such as the prefrontal cortex, four-chambered heart, lungs, and brown adipose tissue are absent (Table 1). Lack of brown adipose tissue in the zebrafish is due to the poikilothermic nature of this organism, which eliminates the need for heat generation (Seth et al., 2013). While lacking lungs, the zebrafish swim bladder shares both anatomical and transcriptional similarities with the mammalian lung, and has been used as an inflammation model in acute lung injury (Zhang et al., 2016). Zebrafish larvae can oxygenate through diffusion alone during the first few days of life, thus allowing the modeling of severe heart defects that cause embryonic lethality in mammals (Asnani and Peterson, 2014). Importantly, despite the absence of a prefrontal cortex and expanded telencephalon, zebrafish are capable of complex behaviors such as reversal learning (Colwill et al., 2005; Parker et al., 2012), long-term social memory (Madeira and Oliveira, 2017), and self-administered opioid seeking (Bossé and Peterson, 2017), supporting the reliance on alternative brain regions and/or pathways to perform executive tasks (Parker et al., 2013).

**TABLE 1:** Notable similarities and differences between major zebrafish organs and human counterparts.

**Major zebrafish organs**	**Notable similarities to humans**	**Notable differences from humans**
Brain	• Major brain regions (i.e., telencephalon, thalamus, cerebellum) are present (Menke et al., 2011) • Dopaminergic, serotonergic, and cholinergic neuronal populations are present (Parker et al., 2013) • Similar myelin structure (Preston and Macklin, 2015)	• Prefrontal cortex and expanded telencephalon are absent (Parker et al., 2013) • Some differences in topography (Parker et al., 2013)
Eyes	• Similar retinal cell layer architecture (Angueyra and Kindt, 2018) • Rods and cones are present, cone-dominant vision (Morris and Fadool, 2005; Chhetri et al., 2014)	• Lateral eyes, spheroid lens (Chhetri et al., 2014) • Also has UV-sensitive cones, tetrachromatic rather than trichromatic vision (Chhetri et al., 2014)
Heart	• First and second heart field progenitor populations have been identified (Asnani and Peterson, 2014) • Similar action potential and electrocardiogram profiles (Verkerk and Remme, 2012; Vornanen and Hassinen, 2016) • Fundamental current systems are present (Vornanen and Hassinen, 2016)	• Two-chambered heart (Asnani and Peterson, 2014) • Different ion channel depolarization/repolarization profiles (Verkerk and Remme, 2012) • Regeneration following partial tissue loss (Poss et al., 2002) • Diffusion-based oxygenation during early development (Asnani and Peterson, 2014)
Kidney	• Glomeruli, proximal/distal tubules, collecting ducts, and brush border membrane are present (Menke et al., 2011; Outtandy et al., 2019) • Major solute transporters and receptors are present (Outtandy et al., 2019)	• Pronephros rather than metanephros (Outtandy et al., 2019)
Liver	• All major mammalian liver cell types are present except Kupffer cells, cell type-specific functions are largely conserved (Goessling and Sadler, 2015; Pham et al., 2017) • Bile secretion *via* bile canaliculi (Goessling and Sadler, 2015; Pham et al., 2017)	• Kupffer cells are not observed (Goessling and Sadler, 2015; Pham et al., 2017) • Different cellular architecture (i.e., hepatocytes arranged into tubules, lobules and portal triads not present; Goessling and Sadler, 2015; Pham et al., 2017)
Pancreas	• Endocrine and exocrine compartments (Seth et al., 2013) • Acinar cells are present; β-, α-, δ-, and ε-cells produce insulin, glucagon, somatostatin, and ghrelin, respectively (Kinkel and Prince, 2009; Seth et al., 2013) • Similar islet architecture (Kinkel and Prince, 2009)	• Less discrete demarcation of organ (Menke et al., 2011)
Adipose tissue	• Multiple white adipose tissue depots, neutral lipid droplets are observable (Zang et al., 2018) • Adipose lineage expresses *pparg* and *fabp4* (Flynn et al., 2009; Zang et al., 2018) • Major metabolic pathways (i.e., SREBFs, PPARs, LEP) are present (Oka et al., 2010; Zang et al., 2018)	• No brown adipose tissue (Zang et al., 2018)
Swim bladder	• Epithelial surfactants are found in swim bladder (Robertson et al., 2014) • Transcriptome shares similarities with mammalian lungs (Zheng et al., 2011; Cass et al., 2013)	• No lungs

### Genetics

The zebrafish reference genome was fully assembled in 2013, and identified 26206 protein-coding genes (Howe et al., 2013). Zebrafish belong to the teleostei infraclass, which has undergone one additional round of whole-genome duplication (teleost-specific genome duplication) relative to other vertebrates (Howe et al., 2013). Over time, the majority of the duplicates (ohnologues) were lost, while those remaining underwent subfunctionalization, neofunctionalization, or function retention (Pasquier et al., 2017). Currently, 26% of all zebrafish genes exist as ohnologue pairs (Howe et al., 2013). Comparison between the zebrafish and human reference genomes revealed 71% of human genes have at least one zebrafish orthologue, and while 69% of zebrafish genes have at least one human orthologue (Howe et al., 2013). Importantly, 82% of all human disease-associated genes have orthologues in the zebrafish (Varga et al., 2018), and supporting the use of the latter in disease modeling.

First demonstrated to be genetically tractable in the 1980s (Howe et al., 2013), the zebrafish system has since proven to be highly amenable toward genetic manipulation. Mutations in zebrafish genes are typically introduced *via* forward or reverse genetics. In forward genetics, random mutations are generated with chemical mutagen or retroviral-mediated DNA insertion, followed by phenotypic screening of the progeny and subsequent genome mapping to isolate the causative locus (Phillips and Westerfield, 2020). Alternatively, under the TILLING (Targeting Induced Local Lesions in Genomes) approach, and genes of interest are screened after the initial mutagenesis (Phillips and Westerfield, 2020). The TILLING method formed the basis of the Zebrafish Mutation Project (Kettleborough et al., 2013) that, together with largescale forward mutagenesis efforts (Driever et al., 1996; Haffter et al., 1996; Amsterdam et al., 1999; Phillips and Westerfield, 2020), and added significantly to the current repertoire of available zebrafish mutants (ZIRC, 2006; CZRC, 2012; EZRC, 2012).

While forward genetics can yield large libraries of mutations that require further genetic characterization, reverse genetics targets known genes of interest. Antisense morpholinos (MOs) are chemically synthesized oligomers that bind specific regions of mRNA to inhibit splicing or translation, resulting in transient protein knockdown without altering DNA sequence (Stainier et al., 2017). While MOs present a valuable tool in studies of early development, stringent guidelines for controls, and rescue experiments must be followed to exclude off-target effects (Stainier et al., 2017). More recently, advancements in targeted gene editing methods such as zinc finger nucleases (Foley et al., 2009), TALENs (Hwang et al., 2014), and CRISPR-Cas9 (Hwang et al., 2013a, b; Gagnon et al., 2014) have ushered in the rapid expansion of stable zebrafish models. CRISPR-Cas9-mediated knockout in the zebrafish is highly efficient [99% mutagenesis success and 28% average germline transmission for an 83-gene panel (Varshney et al., 2015)], thus supporting both single mutation generation and reverse genetics screens.

### Toolkit for Model Characterization

Alongside the rise in zebrafish models, the past decade has also witnessed an expansion in techniques for phenotypic characterization. The zebrafish is routinely processed for *in situ* hybridization (Thisse and Thisse, 2008) and histology (Sullivan-Brown et al., 2011; Copper et al., 2018), and is also amenable to light and electron microscopy (EM; Hildebrand et al., 2017), μCT (Grimes et al., 2016; Ding et al., 2019), and MRI (Koth et al., 2017). High-resolution serial-section EM has been achieved for the entire larval zebrafish brain, allowing visualization of all myelinated axons (Hildebrand et al., 2017). As a potential alternative to traditional histology, 3D reconstruction of whole larval, and juvenile zebrafish from synchrotron-based *X*-ray μCT data was recently obtained at cell resolution, yielding histology-like cross-sections without the need for tissue sectioning (Ding et al., 2019).

Complementing the aforementioned approaches, transgenic zebrafish with cell type-specific fluorescence has become widely adopted in light microscopy. Common transgenic lines such as the Tg(*fli1*:EGFP) allow visualization of the vasculature during early development (Lawson and Weinstein, 2002), while zebrafish expressing yellow fluorescent protein fused to annexin V enables imaging of live apoptotic cells *in vivo* (van Ham et al., 2010). As these fish are genetically stable, they can be crossed into zebrafish disease models to facilitate visualization of cellular changes in the progeny. High-throughput imaging has also been optimized in the larval zebrafish to accommodate fluorescence- (Wang et al., 2015), luminescence- (Mosser et al., 2019), cardiac- (Shin et al., 2010), and behavior-based (Bruni et al., 2016) readouts; implementation of these platforms in high-throughput chemical screens has led to the discovery of compounds that modulate diverse cellular pathways (Liu et al., 2014; Rennekamp et al., 2016; Early et al., 2018).

It is also important to acknowledge the necessity of omics-based technologies for the zebrafish organism, as in-depth knowledge of the zebrafish transcriptome, proteome, and metabolome will not only serve as a valuable resource for the research community, but also be a predictive tool for target selection during model generation. Notably, scGESTALT, which combines the CRISPR-Cas9-based lineage tracing ability of the GESTALT method (McKenna et al., 2016) with single-cell RNA sequencing, enabled the identification of over 100 cell types in the juvenile zebrafish brain (Raj et al., 2018). On the metabolite level, lipidomic analyses of zebrafish larvae have yielded detailed information on hundreds of lipid species and demonstrated the existence of all major mammalian lipid classes in the zebrafish (Fraher et al., 2016; Quinlivan et al., 2017). Conveniently, similarity between the human and zebrafish lipidomes permits the use of existing mammalian lipid databases for processing zebrafish data, thereby eliminating the need for additional curation. Taken together, as zebrafish disease models rise in prevalence, the current toolkit for phenotypic characterization is likely to also expand to match the growing complexity of characterizable phenotypes.

## Zebrafish Models of Lysosomal Storage Diseases

A survey of the existing literature identified 60 zebrafish LSD models with phenotypic data. A list of these models is in Table 2 (see Supplementary Table S1 for additional details). This list was generated based on queries performed in (Pubmed, 1996; search term: zebrafish lysosomal storage disease; last date of query: 1/7/20; ZFIN, 1994; Pubmed, 1996)^1^ [genes from Table 1 of Platt et al. (2018) were searched individually, and all publications under the MUTATIONS AND SEQUENCE TARGETING REAGENTS section and the PHENOTYPE section were evaluated for inclusion; last date of query: 1/9/20]. While we discuss some of these models in subsequent sections, we do not claim our list to be exhaustive and apologize for any omissions made. For the scope of this review we limit our focus to LSD models with published phenotypes that differ from controls. The combination of largescale forward mutagenesis screens (Phillips and Westerfield, 2020) and the Zebrafish Mutation Project (Kettleborough et al., 2013) has yielded an impressive number of characterized and uncharacterized stable mutations in LSD-associated genes, many of which are available through international zebrafish resource centers (ZIRC, 2006; CZRC, 2012; EZRC, 2012).

**TABLE 2:** List of published zebrafish LSD models.

**Gene^§^**	**LSD**	**Zebrafish model**	**References**
**Sphingolipidoses**			
ASAH1*	Farber lipogranulomatosis	Morpholino (*asah1b*)	Zhou et al., 2012
		*asah1a^+68/+68^*; *asah1b^–20/–20^* (CRISPR-Cas9)	Zhang T. et al., 2019
*ARSA*	Metachromatic leukodystrophy	Morpholino	Berg et al., 2016
GALC*	Globoid cell leukodystrophy/Krabbe disease	Morpholino (*galca*)	Zizioli et al., 2014
		Morpholino (*galcb*)	Zizioli et al., 2014
		Morpholino (*galca; galcb*)	Zizioli et al., 2014
*GBA*	Gaucher disease	Morpholino	Zancan et al., 2014
		*gba^*sa1621/sa1621*^* (ENU)	Busch-Nentwich et al., 2012; Zancan et al., 2014
		*gba^*sa391/sa391*^* (TALENs)	Keatinge et al., 2015; Watson et al., 2019
		Morpholino	Berg et al., 2016
		*gba^Δ31/Δ31^* (CRISPR-Cas9)	Lelieveld et al., 2019
*HEXA*	Tay-Sachs disease	Morpholino	Berg et al., 2016
*HEXB*	Sandhoff disease	Morpholino	Kalén et al., 2009
		*herb^–^^14⁣/^^–14^* (CRISPR-Cas9)	Kuil et al., 2019
**Mucolipidoses**			
*GNPTAB*	Mucolipidosis II α/β (I-cell disease); mucolipidosis III α/β (pseudo-Hurler polydystrophy)	Morpholino	Flanagan-Steet et al., 2009, 2016a, 2018; Petrey et al., 2012; Qian et al., 2015
		*gnptab^–/–^* Line 1 (TALENs)	Flanagan-Steet et al., 2018
		*gnptab^–/–^* Line 2 (TALENs)	Flanagan-Steet et al., 2018
*GNPTG*	Mucolipidosis III γ, variant pseudo-Hurler polydystrophy	*gnptg^*zm00105646Tg/zm00105646Tg*^* (DNA insertion)	Flanagan-Steet et al., 2016b
MCOLN1*	Mucolipidosis IV	*mcoln1a^*hg82/hg82*^; mcoln1b^*hg84/hg84*^* (TALENs/CRISPR-Cas9)	Li et al., 2017
		*mcoln1a*^*hg82/hg82*^ (TALENs)	Li et al., 2017
		*mcoln1b*^*hg84/hg84*^ (CRISPR-Cas9)	Li et al., 2017
		*mcoln1a^*hkz13/hkz13*^ (biluo;* ENU)	Jin et al., 2019
**Neuronal ceroid lipofuscinoses**			
*ATP13A2*	CLN12/Kufor-Rakeb syndrome	Morpholino	Lopes da Fonseca et al., 2013
		Morpholino	Spataro et al., 2019
*CLN3*	CLN3/Batten-Spielmeyer-Sjogren disease	Morpholino	Wager et al., 2016
		Morpholino	Wager et al., 2016
*CTSD*	CLN10	Morpholino	Follo et al., 2011; Follo et al., 2013
GRN*	CLN11	Morpholino (*grna*)	Li et al., 2010, 2013
		Morpholino (*grnb*)	Li et al., 2013
		Morpholinos (2 *grna* lines)	Chitramuthu et al., 2010, 2017b
		Morpholino (*grnb*)	Chitramuthu et al., 2010
		Morpholinos (2 *grna* lines, 2 *grnb* lines, 1 *grna; grnb* line)	Laird et al., 2010; De Muynck et al., 2013
*TPP1*	CLN2	*tpp1^*sa11/sa11*^* (ENU)	Busch-Nentwich et al., 2010b; Busch-Nentwich et al., 2013; Mahmood et al., 2013
		*tpp1^*hu3587/hu3587*^* (ENU)	Busch-Nentwich et al., 2010a; Mahmood et al., 2013
		Morpholinos (2 lines)	Mahmood et al., 2013
**Integral membrane protein disorders**			
*CTNS*	Cystinosis	*ctns^*sa14661/sa14661*^* (ENU)	Busch-Nentwich et al., 2013; Elmonem et al., 2017
		Morpholino	Elmonem et al., 2017
		*ctns*^*zh601/zh601*^ (TALENs)	Festa et al., 2018
*NPC1*	Niemann-Pick disease type C1; type D	Morpholinos (2 lines)	Schwend et al., 2011; Louwette et al., 2012; Chu et al., 2015
		*npc1*^*ihb334/ihb334*^ (CRISPR-Cas9)	Lin et al., 2018
		*npc1*^*ihb335/ihb335*^ (CRISPR-Cas9)	Lin et al., 2018
		*npc1*^*hg37/hg37*^ (CRISPR-Cas9)	Tseng et al., 2018
		*npc1*^*y535/y535*^ (CRISPR-Cas9)	Tseng et al., 2018
SCARB2*	Action myoclonus-renal syndrome	*scarb2a^*hi1463Tg/hi1463Tg*^* (DNA insertion)	Golling et al., 2002; Amsterdam et al., 2004; Diaz-Tellez et al., 2016
		*scarb2a*^*hi2715Tg/hi2715Tg*^ (DNA insertion)	Amsterdam et al., 2004
**Glycogen storage disease**			
*GAA*	Pompe disease	*gaa*^*zf752/zf752*^ (TALENs)	Wu et al., 2017
**Glycoproteinosis**			
*MANBA*	β-mannosidosis	Morpholino	Ko et al., 2017
**Mucopolysaccharidosis**			
*IDS*	Mucopolysaccharidosis II/Hunter syndrome	Morpholino	Moro et al., 2010; Costa et al., 2017; Bellesso et al., 2018
		*ids*^*ia200/ia200*^ (CRISPR-Cas9)	Bellesso et al., 2018
**Lysosome-related organelle (LRO) disorders**			
*HSP5*	Hermansky-Pudlak disease type 5	*hsp5^*m454/m454*^ (snow white;* ENU)	Driever et al., 1996; Stemple et al., 1996; Daly et al., 2013
		Morpholino	Daly et al., 2013
HSP7*	Hermansky-Pudlak disease type 7	*dtnbp1a^*ihb819/ihb819*^* (CRISPR-Cas9)	Chen et al., 2018
*LYST*	Chédiak-Higashi disease	*lyst*^*muz107/muz107*^ (ENU)	Kim et al., 2015

^§^Gene denotes human gene names. *Human genes with more than one known orthologue in the zebrafish, the orthologue(s) targeted for mutation are denoted in the names of the zebrafish models.

**FIGURE 1:**
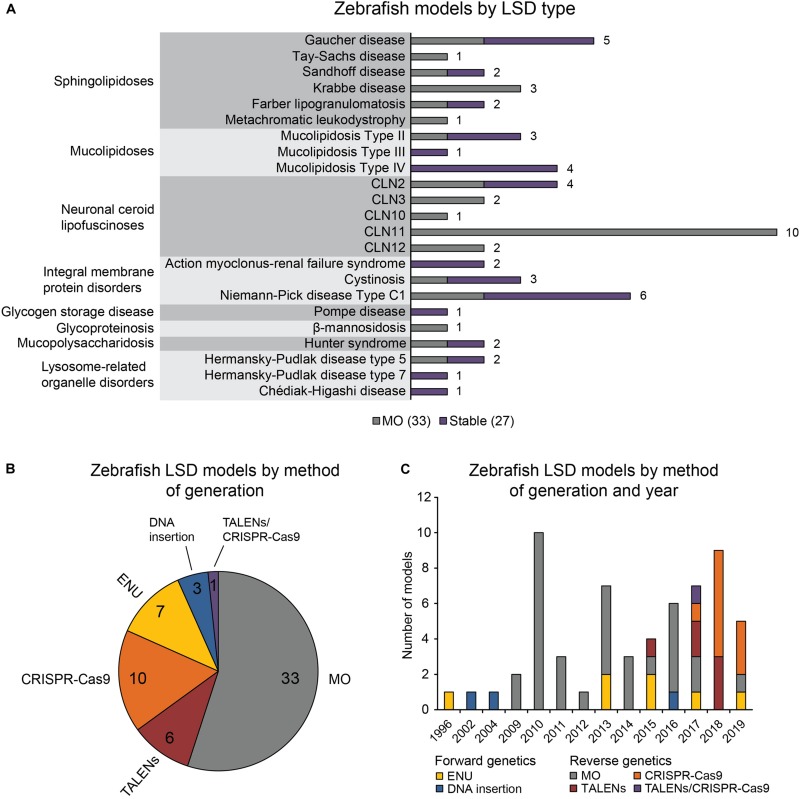
Zebrafish LSD models, a statistical overview. **(A)** Zebrafish LSD models by disease type. Numbers in the bar graph denote total number of published zebrafish models for each LSD. Transient knockdowns (MOs) and stable mutants are indicated. **(B)** Zebrafish LSD models by method of generation. **(C)** Zebrafish LSD models by method of generation and year. Year refers to the earliest year in which phenotypic data were first published.

The 60 zebrafish models could be classified into eight disease categories including sphingolipidoses (14), MLs (8), neuronal ceroid lipofuscinoses (19), integral membrane protein disorders (11), glycogen storage disease (1), glycoproteinosis (1), MPS (2), and lysosome-related organelle disorders (4; Figure 1A). (Platt et al., 2018) 55% (33) of the models are transient MO knockdowns, while the rest (27) are stable mutants (Figures 1A,B). Stable mutants were generated using both forward [N-ethyl-N-nitrosourea (ENU): 7, DNA insertion: 3], and reverse genetics (TALENs: 6, CRISPR-Cas9: 10; combined TALENs/CRISPR-Cas9: 1) approaches (Figure 1B). Notably, organization of the reported models by method of generation and year revealed a relatively large number of transient knockdowns MOs up to 2016, which gradually shifted toward stable TALEN and CRISPR-Cas9 models from 2017 to 2019 (Figure 1C). This occurrence is a likely reflection of the successful implementation of these gene editing methods in the zebrafish starting from 2011 (Sander et al., 2011; Hwang et al., 2013a, b, 2014; Gagnon et al., 2014). In the following subsections we discuss the aforementioned zebrafish LSD models in detail.

### Zebrafish Models of Sphingolipidoses

Sphingolipidoses are a subcategory of LSDs that affect the sphingolipid catabolic pathway (Özkara, 2004). Loss-of-function mutations in enzymes of sphingolipid catabolism lead to sphingolipid accumulation, and a spectrum of LSD pathologies that can affect both the CNS and peripheral organs (Özkara, 2004). Sphingolipids form an essential lipid class that regulates all major aspects of cell biology, and early disruptions in sphingolipid metabolism are associated with embryogenesis defects (Herr et al., 2003; Wang and Bieberich, 2017). Sphingolipid metabolism is highly conserved across species and centers around ceramide, the core of the sphingolipid pathway through which additional sphingolipids undergo interconversions (Futerman and Riezman, 2005).

**FIGURE 2:**
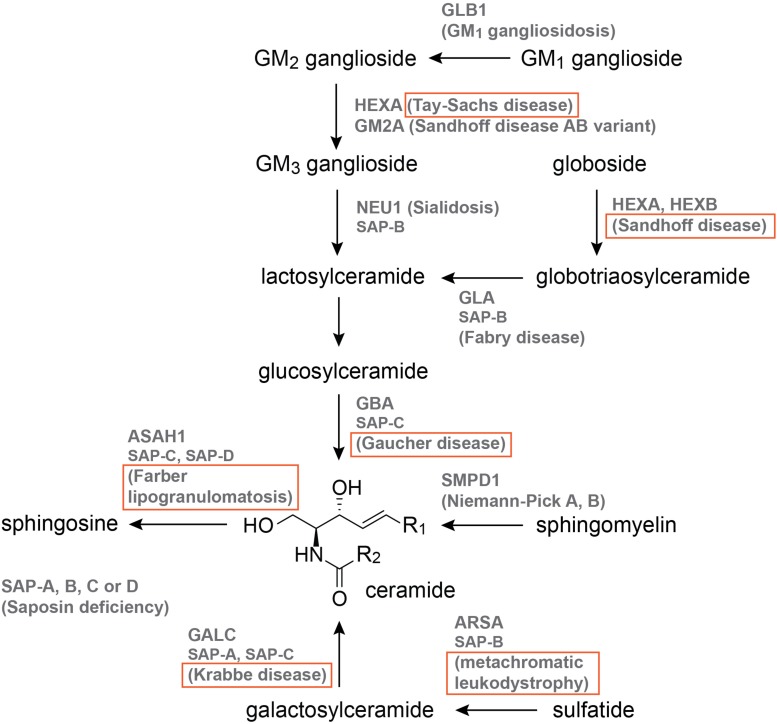
The sphingolipid catabolic pathway. Names that are boxed have published zebrafish models. Pathway illustration is based on Özkara (2004).

The 12 sphingolipid-associated LSDs are illustrated in Figure 2, and those with published zebrafish models are boxed. Of these models, nine are MO knockdowns, three are generated by CRISPR-Cas9, one is generated by TALENs, and one derives from the Zebrafish Mutation Project (Kettleborough et al., 2013), which used the chemical mutagen N-ENU in its forward genetics phase (Figure 2, Table 2, and Supplementary Table S1). The published models are for Gaucher disease (5; Zancan et al., 2014; Keatinge et al., 2015; Lelieveld et al., 2019), Krabbe disease (3; Zizioli et al., 2014), Tay-Sachs disease (1; Berg et al., 2016), Sandhoff disease (2; Kalén et al., 2009; Kuil et al., 2019), metachromatic leukodystrophy (1; Berg et al., 2016), and Farber lipogranulomatosis (2; Zhang T. et al., 2019; Zhou et al., 2012; Figure. 2, Table 2, and Supplementary Table S1).

Five zebrafish models focused on Gaucher disease, which is caused by mutations in β-glucocerebrosidase (GBA), an enzyme responsible for lysosomal glucosylceramide breakdown (Figure 2; Platt et al., 2018). Gaucher disease is classified into non-neuropathic Type I, and neuropathic Types II and III (Genetics Home Reference, 2003). Given the presentation of skeletal abnormalities in Type I Gaucher disease patients, Zancan et al. used MO and a stable mutant (*gba*^*sa1621/sa1621*^, Table 2, and Supplementary Table S1) from the Zebrafish Mutation Project to investigate bone development (Kettleborough et al., 2013; Zancan et al., 2014). *gba* morphants exhibited reduced expression of early bone development markers, decreased ossification, anemia, hepatomegaly, and impaired canonical Wnt signaling, which were recapitulated by the stable mutant (Zancan et al., 2014). Impaired Wnt signaling was also present in fibroblasts isolated from type I Gaucher disease patients, and the reduced Wnt signaling in Gba-deficient zebrafish could be rescued *via* injection of *GBA* mRNA (Zancan et al., 2014). A second stable Gaucher disease model (*gba*^*sh391/sh391*^, Table 2, and Supplementary Table S1) was generated by Keatinge et al. (2015) using TALENs and subjected to extensive phenotypic characterization with a focus on the CNS. Pathologies associated with the *gba*^*sh391/sh391*^ zebrafish include impaired locomotion, sphingolipid accumulation, microglial activation, and mitochondrial dysfunction; in the CNS, dopaminergic neuron loss and ubiquitylated neuronal cytoplasmic inclusions were identified at 12 weeks-post-fertilization (wpf), and no survival was recorded past 14 wpf (Keatinge et al., 2015). More recently, glucosylsphingosine accumulation was demonstrated in a CRISPR-Cas9 model of Gba deficiency (*gba^Δ31/Δ31^*, Table 2, and Supplementary Table S1), which could be rescued by overexpression or infusion of human GBA (Lelieveld et al., 2019).

One MO was reported for Tay-Sachs disease, and one MO (Kalén et al., 2009) and one CRISPR-Cas9 model (Kuil et al., 2019) for Sandhoff disease. Tay-Sachs and Sandhoff disease are GM_2_ gangliosidoses that severely affect the CNS (Platt et al., 2018). Sandhoff disease is caused by mutations in the *HEXB* gene that encodes the β-subunit of either the β-hexosaminidase (HEXB: two β-subunits) or α-hexosaminidase (HEXA: one β- and one α-subunit) enzyme, while Tay-Sachs disease involves mutations in *HEXA*, which encodes the α-subunit of HEXA (Genetics Home Reference, 2003). The *hexb* morphant was generated as part of a reverse genetics screen for angiogenesis modulators, and was thus characterized with a focus on the vasculature (Kalén et al., 2009). A fraction of *hexb* morphants exhibited intersegmental vessel defects at the 48–56 hours-post-fertilization (hpf) stage (Kalén et al., 2009). More recently, a CRISPR-Cas9 *hexb* model (*hexb^–14/–14^*, Table 2, and Supplementary Table S1) was generated by Kuil et al. (2019) and evaluated for perturbations in cells of the CNS. Larval pathologies include altered microglial lysosome morphology, increased radial glial lysosome speckles, increased late apoptotic cells in the brain, and reduced locomotion (Kuil et al., 2019). Surprisingly, despite significant oligosaccharide accumulation in the brain and peripheral organs, adult *hexb^–14/–14^* fish are viable without obvious swim defects (Kuil et al., 2019). Morphants for *hexa* were generated in the context of infectious disease research, and were used to pinpoint lysosomal storage as a contributor toward impaired macrophage migration and increased tuberculosis (TB) susceptibility (Berg et al., 2016). *hexa* morphants contained macrophages with enlarged lysosomes and reduced migration speed, and were more susceptible to TB infection; additional MOs against *gba* (Gaucher disease) and *arsa* (metachromatic leukodystrophy) revealed similar alterations in macrophage movement and TB susceptibility (Berg et al., 2016).

Of the 12 known sphingolipidoses-associated genes, *ASAH1*, and *GALC* are duplicated in zebrafish (Frankish et al., 2017). Mutations in *ASAH1* (acid ceramidase) and *GALC* (galactosylceramidase) lead to Farber lipogranulomatosis and Krabbe disease, respectively (Platt et al., 2018). As the functional status of uncharacterized ohnologues is not always bioinformatically predictable, the generation of both single and double mutants may be necessary to confirm the anticipated ohnologue function. Using a combination of activity assays, database query, and cloning, Zizioli et al. (2014) characterized *galca* and *galcb* as the orthologues of GALC. While MO against *galca* or *galcb* alone only partially reduced Galc activity and mildly altered expression of the transcription factor *neuroD*, co-injection of *galca* and *galcb* MOs resulted in complete loss of Galc activity, reduced and partially disorganized *neuroD* pattern, and increased apoptosis (Zizioli et al., 2014). Similar to GALC, two zebrafish orthologues (Asah1a, Asah1b) correspond to ASAH1. MO knockdown of Asah1b led to reduced motor neuron axonal branching and increased apoptosis in the spinal cord without gross developmental delays (Zhou et al., 2012). *asah1a/b^–/–^* (*asah1a^+68/+68^*; *asah1b^–20/–20^*, Table 2, and Supplementary Table S1) zebrafish has also been generated using CRISPR-Cas9, and exhibited reduced size, ceramide accumulation, and early mortality [4 months-post-fertilization (mpf); Zhang X. et al., 2019]. No changes in size, ceramide content, or lifespan were observed in Asah1 single mutants, suggesting that Asah1a and Asah1b possess some functional redundancy (Zhang T. et al., 2019). More detailed phenotypic characterization of Asah1 mutants could shed further light on the functional impacts unique to each ohnologue.

### Zebrafish Models of Mucolipidoses

Mucolipidoses are characterized by cellular storage of carbohydrates, proteins and/or lipids that leads to a spectrum of symptoms ranging from mild intellectual delay to severe ataxia and congestive heart failure (Mucolipidosis Fact Sheet, 2020). Symptom onset ranges from infantile to adult (Platt et al., 2018). MLs are classified into five types (ML I, ML II α/β (I-cell disease), ML III α/β (pseudo-Hurler polydystrophy), ML III γ, and ML IV) based on the affected enzyme (Genetics Home Reference, 2003; Platt et al., 2018). ML II α/β and ML III α/β are caused by mutations in the same enzyme, but vary in symptoms and times of onset (Genetics Home Reference, 2003).

Eight ML models have been characterized for ML II α/β and ML III α/β (MO, TALENs; Flanagan-Steet et al., 2009; Petrey et al., 2012; Qian et al., 2015; Flanagan-Steet et al., 2016a; Flanagan-Steet et al., 2018), ML III γ (DNA insertion; Flanagan-Steet et al., 2016b), and ML IV (TALENs/CRISPR-Cas9, TALENs, and ENU; Li et al., 2017; Jin et al., 2019). ML II α/β and ML III α/β are caused by mutations in *GNPTAB*, which encodes the α- and β-subunits of N-acetylglucosamine-1-phosphotransferase, a heterotrimeric enzyme that catalyzes the biosynthesis of mannose-6-phosphate markers required for the accurate targeting of lysosomal enzymes (Flanagan-Steet et al., 2009). Flanagan-Steet et al. (2009) designed two MOs targeting *gnptab*. Major pathologies associated with *gnptab* morphants include impaired motility, craniofacial and otic vesicle defects, and dysregulated chondrocyte differentiation program as evidenced by abnormal Sox9 and type II collagen expression (Flanagan-Steet et al., 2009). Continued investigation of *gnptab* morphants revealed imbalanced TGF-β/BMP signaling that was potentiated by sustained activation of cathepsin K (Ctsk; Petrey et al., 2012; Flanagan-Steet et al., 2016a). Intriguingly, TGF-β and Ctsk activation appears to be reciprocal, as TGF-β also enhanced Ctsk processing by elevating chondroitin-4-sulfate (C4-S; Flanagan-Steet et al., 2018). Inhibition of TGF-β, Ctsk, or *chst11* (C4-S biosynthesis enzyme) each rescued the craniofacial defects found in *gnptab* morphants (Petrey et al., 2012; Flanagan-Steet et al., 2018). Taken together, these findings support a mechanism for the perturbed chondrogenesis in Gnptab deficiency in which TGF-β signaling raises C-S4 to drive Ctsk activity, which then further activates TGF-β signaling (Flanagan-Steet et al., 2018). Unlike *gnptab* morphants, craniofacial and cathepsin changes were not present in an insertional mutant of *gnptg* (γ-subunit of N-acetylglucosamine-1-phosphotransferase), although delayed development and reduced survival were observed in *gnptg^–/–^* embryos (Flanagan-Steet et al., 2016b).

Stable genetic models have been reported for ML IV, which is caused by mutations in mucolipin I (*MCOLN1*), a lysosomal cation channel of the Transient Receptor Potential family (Li et al., 2017). *MCOLN1* is duplicated in the zebrafish (*mcoln1a*, *mcoln1b*; Frankish et al., 2017). Using TALENs and CRISPR-Cas9, Li et al. (2017) generated zebrafish carrying mutations in both *mcoln1a* and *mcoln1b* (*mcoln1a*^*hg82/hg82*^; *mcoln1b*^*hg84/hg84*^, Table 2, and Supplementary Table S1). In agreement with ML IV pathologies of the muscle and eye, abnormal cell morphologies and autophagosome accumulation were found in the muscle fibers of adult *mcoln1a/b^–/–^* fish, while storage materials and cell loss were present in the *mcoln1a/^–/–^* larval retina (Li et al., 2017). The majority of the reported pathologies were associated with the double, but not single mutant, suggesting that Mcoln1 ohnologues are to some extent functionally redundant (Li et al., 2017). A second hypomorphic mutant, *biluo* (*mcoln1a*^*hkz13/hkz13*^, Table 2, and Supplementary Table S1), was isolated from a forward genetics screen (Jin et al., 2019). Imaging of *biluo* microglia revealed abnormal morphology and phagosome accumulation; late endosome-lysosome fusion was impaired, likely due to the reduced calcium efflux of mutant Mcoln1a (Jin et al., 2019). Excessive spontaneous neuronal activity was observed in the *biluo* optic tectum, which was fully rescued upon wildtype *mcoln1a* expression in the neurons and microglia (Jin et al., 2019).

### Zebrafish Models of Neuronal Ceroid Lipofuscinoses

Lipofuscinoses are characterized by cellular storage of lipofuscin, an autofluorescent pigment composed of oxidized cross-linked macromolecules that increases in content with age (Moreno-García et al., 2018). Lipofuscinoses are divided into fourteen types (CLN1-14) based on the enzyme, symptoms, and times of onset (Platt et al., 2018). Nineteen zebrafish models have been characterized for five lipofuscinoses, including CLN2 (MOs, ENU; Mahmood et al., 2013), CLN3 (MOs; Wager et al., 2016), CLN10 (MO; Follo et al., 2011; Follo et al., 2013), CLN11 (MOs; Chitramuthu et al., 2010, 2017b; Laird et al., 2010; Li et al., 2010, 2013; De Muynck et al., 2013), and CLN12 (MOs; Lopes da Fonseca et al., 2013; Spataro et al., 2019).

CLN2, or Jansky-Bielschowsky disease, is caused by mutations in lysosomal serine protease tripeptidyl peptidase 1 (TPP1; Mahmood et al., 2013). In agreement with the severe CNS pathologies found in many CLN2 patients, phenotypic characterization of a *tpp1* zebrafish mutant (*tpp1*^*sa11/sa11*^, Table 2, and Supplementary Table S1) from the Zebrafish Mutation Project revealed gross morphological abnormalities and early-onset neurodegeneration that led to death by 5 days-post-fertilization (dpf; Kettleborough et al., 2013; Mahmood et al., 2013). Neuron loss, increased apoptosis, and storage material accumulation were detected in the optic tectum, cerebellum, and retina of *tpp1*^*sa11/sa11*^ larvae; CNS cell proliferation and axon guidance were disrupted in both *tpp1*^*sa11/sa11*^ larvae and *tpp1* morphants (Mahmood et al., 2013). Behaviorally, *tpp1*^*sa11/sa11*^ larvae exhibited longer movement bouts and repetitive twitching indicative of seizures (Mahmood et al., 2013). Similar to *tpp1*^*sa11/sa11*^ larvae, gross morphological abnormalities, and neurodegeneration were also present in *cln3* (Batten disease) morphants (Wager et al., 2016). *cln3* morphant heart was elongated and lacked pigmentation, and EEG recordings of the optic tectum demonstrated epileptiform activity (Wager et al., 2016). In agreement with the storage of mitochondrial ATP synthase subunit c (SCMAS) in CLN2 and CLN3, SCMAS accumulation was observed in both *tpp1*^*sa11/sa11*^ larvae and *cln3* morphants (Wager et al., 2016).

CLN10 and CLN11 are caused by mutations in *ctsd* (cathepsin D) and *grn* (progranulin), respectively (Platt et al., 2018). Cathepsin D (CD) is an endosomal/lysosomal aspartic protease responsible for intracellular proteolysis (Follo et al., 2011). MO knockdown of CD led to impaired yolk absorption, hyperpigmentation, absence of swim bladder, decreased survival, and morphological abnormalities of the somatic musculature and retinal pigmented epithelium (Follo et al., 2011; Follo et al., 2013). Progranulin is a secreted glycoprotein containing seven and a half non-identical tandem repeats of the 12-cysteine granulin motif; proteolytic cleavage of progranulin generates granulin peptides that regulate diverse pathways including wound healing, inflammation, and lysosome homeostasis (Bhandari et al., 1992; Chitramuthu et al., 2017a). *GRN* is duplicated in the zebrafish and MOs have been designed against *grna* and/or *grnb* (Chitramuthu et al., 2010, 2017b; Laird et al., 2010; Li et al., 2010, 2013; De Muynck et al., 2013). Using MOs targeting *grna*, Li et al. (2010, 2013) observed impaired liver and muscle growth, as evidenced by decreased hepatocyte and myogenic progenitor cell (MPC) proliferation, decreased liver size, and abnormal myofiber morphology. Grna regulation of liver outgrowth and myogenesis occurs partly through modulation of Met signaling, a known regulator of hepatic and MPC expansion (Li et al., 2010; Li et al., 2013). In the CNS, *grna* knockdown led to caudal primary motor neuron (CaP MN) truncation, premature branching, and stalled outgrowth (Chitramuthu et al., 2010, 2017b). The clustering of nicotinic acetylcholine receptors, a key prerequisite for MN development, appeared mislocalized and disorganized (Chitramuthu et al., 2017b). As a likely consequence of the observed MN defects, touch response was decreased in *grna* morphants (Chitramuthu et al., 2017b).

Importantly, mutations in LSD-associated genes have also been implicated in additional neurodegenerative disorders. While the majority of LSDs are autosomal recessive (Platt et al., 2018), increased incidence of Parkinson’s disease has been observed in both Gaucher disease patients and asymptomatic carriers (Riboldi and Di Fonzo, 2019). Mutations in progranulin cause frontotemporal lobar degeneration (FTLD), and progranulin exerts neuroprotective roles against TAR DNA-binding protein 43 aggregation, the latter a feature of amyotrophic lateral sclerosis (ALS) and FTLD (Chitramuthu et al., 2010, 2017b; Laird et al., 2010). Mutations in the lysosomal transmembrane ATPase ATP13A2, the causal enzyme behind CLN12, have been linked to familial cases of Parkinson’s disease (Lopes da Fonseca et al., 2013) and juvenile-onset ALS (Spataro et al., 2019). Consistent with Parkinson’s disease and ALS pathologies, MO-induced partial Atp13a2 knockdown led to locomotor impairment, disorganized cerebellar axons, and defective MN path finding (Lopes da Fonseca et al., 2013; Spataro et al., 2019). As additional mutations in LSD genes are uncovered, targeted gene editing with finetuned control over the mutations of interest will enable more precise disease modeling.

### Zebrafish Models of Integral Membrane Protein Disorders

Integral membrane protein disorders are so named given their involvement of proteins that localize non-transiently to a cell membrane. The six known integral membrane protein disorders are Danon disease lysosomal associated membrane protein-2 (*LAMP2*), sialic acid storage disease (*SLC17A5*), mucolipidosis IV (*MCOLN1*), Niemann-Pick disease type C1 and C2 (*NPC1* and *NPC2*), cystinosis (*CTNS*), and action myoclonus-renal failure (AMRF) syndrome (*SCARB2*; Platt et al., 2018). Of these, the last four have published zebrafish models. Eleven zebrafish models have been characterized for Niemann-Pick C1 (MOs, CRISPR-Cas9; Schwend et al., 2011; Louwette et al., 2012; Chu et al., 2015; Lin et al., 2018; Tseng et al., 2018), cystinosis (ENU, MOs, TALENs; Elmonem et al., 2017; Festa et al., 2018), and AMRF (DNA insertion; Golling et al., 2002; Amsterdam et al., 2004; Diaz-Tellez et al., 2016). Mucolipidosis IV was discussed earlier in this review.

Two MO and four CRISPR-Cas9 models of Niemann-Pick disease type C1 (NPC1) have been characterized. NPC1 and 2 are caused by mutations in the cholesterol transporters NPC1 and NPC2, resulting in accumulation of cholesterol and additional lipids (Genetics Home Reference, 2003). A missense mutation in *NPC1* has also been identified in Niemann-Pick disease type D, a variant of NPC1 (Greer et al., 1998). Two additional Niemann-Pick disease types, A and B, are caused by mutations in acid sphingomyelinase of the sphingolipid catabolic pathway (Figure 2; Genetics Home Reference, 2003). MO-induced Npc1 knockdown resulted in early (within 1 dpf) developmental defects including delayed epiboly, disorganized actin cytoskeleton, a shorter and wider body axis, and increased cell death (Schwend et al., 2011). Further investigation of *npc1* morphants revealed thrombocytopenia and mild anemia, in accord with the hematological presentation found in some NPC1 patients (Louwette et al., 2012). All of the four published CRISPR-Cas9 *npc1* models contain premature stop codons, with three of the mutations in exon 2, and the fourth one in exon 7 (Table 2 and Supplementary Table S1; Lin et al., 2018; Tseng et al., 2018). Major pathologies associated with *npc1^–/–^* zebrafish include decreased size and survival, lipid storage, vacuolated hepatocytes, impaired swimming, and Purkinje cell defects (Lin et al., 2018; Tseng et al., 2018). Observed phenotypes were largely consistent across CRISPR-Cas9 models.

Five zebrafish models have been characterized for AMRF and cystinosis. AMRF is caused by mutations in *SCARB2*, which encodes LIMP-2, a lysosomal integral membrane protein with various functions including receptor recognition of phospholipids and viruses, cholesterol transport, and the transport of β- GBA (mutated in Gaucher disease) to the lysosome (Genetics Home Reference, 2003; Heybrock et al., 2019). Three zebrafish orthologues (*scarb2a, b, c*) correspond to *SCARB2* (Frankish et al., 2017). Two *scarb2a* insertional mutants (*scarb2a*^*hi1463Tg/hi1463Tg*^, *scarb2a*^*hi2715Tg/hi2715Tg*^, Table 2, and Supplementary Table S1) have been isolated from a largescale forward mutagenesis screen; initial characterization identified hypopigmentation and morphological abnormalities in the hindbrain and notochord (Golling et al., 2002; Amsterdam et al., 2004). Investigation of notochord architecture in the homozygous *hi1463Tg* line revealed incorrectly sized notochord vacuoles, and assembly defects in the notochord-surrounding basement membrane (Diaz-Tellez et al., 2016). Cystinosis is caused by mutations in the transporter protein cystinosin (Ctns) that lead to cystine accumulation (Genetics Home Reference, 2003). Consistent with known cystinosis pathologies, cystine accumulation and kidney defects were observed in a *ctns^–/–^* (*ctns*^*sa14661/sa14661*^, Table 2, and Supplementary Table S1) line from the Zebrafish Mutation Project (Kettleborough et al., 2013; Elmonem et al., 2017). Cystine accumulated in both *ctns*^*sa14661/sa14661*^ larvae (6 dpf) and adults (8 mpf; Elmonem et al., 2017). Kidney pathologies appeared during the adult stage (3–6 mpf), and included enlarged lysosomes, partial podocyte foot process effacement, and compromised glomerulus and proximal tubule function (Elmonem et al., 2017). Increased LC3-II level and autophagic vesicles were also observed in the pronephric tubules of a TALEN (*ctns*^*sa14661/sa14661*^, Table 2, and Supplementary Table S1) mutant generated by Festa et al. (2018). Using primary proximal tubule cells from *ctns^–/–^* mice, the authors identified a link between cystinosis-induced autophagy and epithelial dysfunction in which autophagy-induced mitochondrial oxidative stress activates the transcription factor ZONAB *via* the Gα12/Src/ZO-1 axis, resulting in transcriptional changes that contribute to epithelial dysfunction (Festa et al., 2018).

### Zebrafish Model of Glycogen Storage Disease

Glycogen storage diseases are caused by mutations in non-lysosomal and lysosomal enzymes of glycogen metabolism (Kishnani et al., 2014). Of the 17 known GSDs (Sara and Alan, 2018), Pompe disease (GSD II) and Danon disease (also classifiable as an integral membrane protein disorder) are categorized as LSDs given their involvement of the lysosomal acid α-glucosidase (GAA) and LAMP2, respectively (Platt et al., 2018). While multiple organs are affected, muscle weakening is a common feature of both LSDs, and can lead to heart failure in severe cases (Platt et al., 2018). Using TALENs, Wu et al. (2017) generated an early-termination zebrafish model (*gaa*^*zf752/zf752*^, Table 2, and Supplementary Table S1) of Pompe disease. Adult *gaa*^*zf752/zf752*^ zebrafish exhibited glycogen storage in the liver, heart, and skeletal muscle without phenocopying the severe pathologies of classic infantile-onset Pompe disease, likely due to the preservation of residual Gaa enzyme activity (Wu et al., 2017). It is important to note that similar to mammalian systems, disease modeling in the zebrafish has revealed an absence of expected phenotypes in some instances. A more detailed discussion of this phenomenon is presented in the *Finetuning CRISPR-Cas9 mutagenesis in the zebrafish* subsection.

### Zebrafish Model of Glycoproteinosis

Glycoproteinoses are mainly caused by mutations in enzymes that degrade the oligosaccharides linked to glycoproteins, resulting in the storage of various protein- and oligosaccharide-based materials (Platt et al., 2018). Glycoproteinoses include α-mannosidosis (*MAN2B1*), β-mannosidosis (*MANBA*), fucosidosis (*FUCA1*), aspartylglucosaminuria (*AGA*), Schindler disease (*NAGA*), sialidosis (*NEU1*, also implicated in sphingolipid catabolism, Figure 1), and galactosialidosis (*CTSA*; Platt et al., 2018). Of these, a MO model of β-mannosidosis has been generated in the zebrafish (Ko et al., 2017). β-Mannosidase (MANBA) is the final exoglycosidase in the catabolism of N-linked glycoprotein oligosaccharides (Alkhayat et al., 1998). Using expression quantitative trait loci analysis of human kidney samples, Ko et al. (2017) identified *MANBA* as a potential target gene for chronic kidney disease. In agreement with this finding, *manba* morphants exhibited pericardial edema and renal tubule defects as demonstrated *via* reduced pronephros-specific GFP expression (Ko et al., 2017). Given the multiple-organ pathologies associated with β-mannosidosis, further examination of both transient and stable *manba* models could yield additional phenotypic details.

### Zebrafish Models of Mucopolysaccharidoses

Mucopolysaccharidoses are characterized by cellular accumulation of sulfated and non-sulfated glycosaminoglycans, due to mutations in enzymes that catabolize these species (Platt et al., 2018). MPS are divided into 11 types (MPS I, II, IIIA-D, IVA-B, VI, VII, and IX) based on the affected enzyme (Platt et al., 2018). Hunter syndrome (MPS II) involves iduronate 2-sulfatase (IDS), a key enzyme of the lysosomal dermatan and heparan sulfate degradation pathway (Demydchuk et al., 2017; Platt et al., 2018). Given the necessity of dermatan and heparan sulfate for major cellular processes such as growth and signaling (Trowbridge and Gallo, 2002), excessive accumulation of these polysaccharides leads to multiple-organ pathologies including hepatosplenomegaly, respiratory infection, joint deformation, and heart valve abnormalities (Genetics Home Reference, 2003). In agreement with MPS II clinical presentations, MO knockdown of Ids led to increased mortality, impaired early development, abnormal heart morphogenesis, and craniofacial defects in zebrafish larvae (Moro et al., 2010; Costa et al., 2017). Scoliosis and kyphosis were also identified in 15-mpf CRISPR-Cas9 mutant (*ids*^*ia200/ia200*^, Table 2, and Supplementary Table S1) fish (Bellesso et al., 2018). Importantly, several major signaling pathways were dysregulated in *ids* morphants across different timeframes, including TGF-β, Shh, canonical Wnt, and FGF. Altered Shh and Wnt signaling were associated with defects in heart morphogenesis, while impaired FGF signaling was identified in the context of bone development (Moro et al., 2010; Costa et al., 2017; Bellesso et al., 2018). As further evidence for the role of Shh signaling in heart development in the context of MPS II, embryo treatment with the Smoothened agonist purmorphamine partially rescued the cardiac trabeculation defect associated with Ids knockdown (Costa et al., 2017).

### Zebrafish Models of Lysosome-Related Organelle Disorders

Lysosome-related organelles (LROs) are vesicles that share lysosomal features such as pH and membrane protein components, but also exert distinct, cell type-specific functions (Huizing et al., 2008). Unique LRO populations have been discovered across a wide range of cell types including melanocytes, lymphocytes, lung type II epithelial cells, osteoclasts, and neurons (Huizing et al., 2008). LROs share a common biogenesis machinery, and loss-of-function mutations in LRO biogenesis enzymes lead to LSD-like pathologies (Huizing et al., 2008). LRO disorders are classified into 12 types (HPS1-9; Griscelli syndrome 1, 2; and Chédiak-Higashi disease) based on the affected enzyme (Platt et al., 2018). Oculocutaneous albinism and increased bruising are common clinical features of HPS1-9, while hypopigmentation, recurrent infections, and neurological involvement are found in Griscelli syndrome and Chédiak-Higashi disease (Platt et al., 2018).

Three zebrafish models [ENU (Daly et al., 2013), CRISPR-Cas9 (Chen et al., 2018), and MO (Kim et al., 2015)] have been generated for LRO disorders. An ENU-generated zebrafish model of HPS5, *snow white* (*snw*, *hsp5*^*m454/m454*^, Table 2, and Supplementary Table S1), was identified from a largescale mutagenesis screen based on its phenotypes of hypopigmentation and abnormal notochord cell morphology (Stemple et al., 1996). Consistent with the presentation of oculocutaneous albinism in HPS patients, *snw* melanosomes exhibited reduced number and size, mislocalization, and impaired maturation (Daly et al., 2013). Lysosomal trafficking regulator (LYST) is the transport protein implicated in Chédiak-Higashi disease, and the *lyst*^*muz107/muz107*^ (Table 2 and Supplementary Table S1) zebrafish was isolated from a forward genetic screen for inherited liver disease, which identified hepatic steatosis and hepatomegaly in a zebrafish mutant that contained a point mutation in the *lyst* locus (Kim et al., 2015). Mutations in dysbindin [DTNBP1, Dtnbp1a, and Dtnbp1b in zebrafish (Frankish et al., 2017)] lead to HPS7, and a CRISPR-Cas9-generated *dtnbp1a* mutant (*dtnbp1a*^*ihb819/ihb819*^) exhibited reduced iridophore reflection, decreased melanin level, poor growth, and compromised survival (Chen et al., 2018). Importantly, dystrophin is one of the ten proteins that form Biogenesis of Lysosome-Related Organelles Complex 1 (BLOC-1), and mutations of other BLOC-1 proteins in the zebrafish also lead to HPS-like pathologies, supporting the existence of additional LSD gene targets (Chen et al., 2018).

## Limitations and Potential Solutions

While the zebrafish model organism confers unique advantages that promote applications otherwise challenging in mammals, there remain limitations that need to be addressed to ensure the success of future model generation and drug discovery efforts. We discuss current limitations and potential solutions associated with model generation, imaging, and chemical screening in the following subsections.

### Finetuning CRISPR-Cas9 Mutagenesis in the Zebrafish

As illustrated in Figure 1C, advancements in targeted gene editing have contributed to the recent rise in reverse genetics models, with some of the most recent zebrafish LSD models being generated *via* CRISPR-Cas9. Originating from bacteria, the CRISPR-Cas9 system employs sequence-specific RNA guides to direct Cas9 cutting of the host DNA at a predetermined site (Cornet et al., 2018). Given the error-prone nature of host cell DNA repair mechanisms such as non-homologous end joining (NHEJ), repair of the Cas9-induced lesion often leads to insertions and/or deletions (INDELs) that may disrupt protein function (Cornet et al., 2018). While Cas9 protein and guides can be delivered easily into zebrafish embryos to create INDELs with high efficiency of germline transmission (Varshney et al., 2015), current limitations include genome duplication, compensation, and mutagenesis precision.

Due to teleost-specific genome duplication (TGD), 26% of all zebrafish genes exist as ohnologue pairs (Howe et al., 2013). Of the 23 LSD-associated human genes with zebrafish models, six (26%) have more than one orthologue in the zebrafish (Table 2 and Supplementary Table S1). For Asah1 (Farber lipogranulomatosis) and Mcoln1 (mucolipidosis IV), where isogenic single and double mutants are both available (Li et al., 2017; Zhang T. et al., 2019), severe pathologies were associated only with the double mutant, suggesting that at least a portion of ohnologues share similar functions. Consequently, TGD adds additional complexity to the zebrafish genome, and necessitates the generation of double mutants in instances of functional redundancy. While the high efficiency of CRISPR-Cas9 gene editing allows mutagenesis of multiple loci *via* co-injection of different RNA guides (Zhang T. et al., 2019), genotyping becomes more challenging as only 6.25% of each doubly heterozygous parent incross is predicted to be doubly mutant progeny. Recent innovations in genotyping technologies (addressed in the next subsection) could accelerate mutation identification and propagation.

As greater numbers of stable zebrafish mutants undergo characterization, it has also become apparent that a portion of these models do not phenocopy the severe pathologies of the targeted disease, and demonstrate residual protein activity even in cases of early-terminating mutations (El-Brolosy and Stainier, 2017; Balciunas, 2018). This phenomenon has been attributed to compensation either through the mutated gene (e.g., alternative start codon, cryptic splice sites and alternative splicing, ribosomal frameshifting, and nonsense readthrough) or through the upregulation of related genes (transcriptional adaptation; El-Brolosy and Stainier, 2017; Balciunas, 2018; El-Brolosy et al., 2019). In instances where alternative splicing or ATG sites are known, evaluation of mutations targeting different exons of the same gene could increase the chance of detecting phenotypes. Notably, using zebrafish and mouse models, El-Brolosy et al. (2019) demonstrated that for the genes investigated, transcriptional adaption is largely triggered by mRNA decay, such that transcription-hindering mutations (i.e., deletions of the promoter region or entire gene locus) failed to trigger transcriptional adaption and led to more severe phenotypes. These findings yield additional insights on the mechanisms underlying transcriptional adaption and suggest alternative guide design strategies toward alleviating this effect.

Given variations in existing LSD mutations and their association with different symptoms, the introduction of LSD-specific mutations into the zebrafish could enable more nuanced phenotype modeling. While INDELs are routinely generated with high efficiency for knockout models, they do not allow control over the mutagenized sequence (Cornet et al., 2018). While less common, TALEN- and CRISPR-Cas9-induced knockins have been achieved by exploiting either the Homology-Directed Repair (HDR) or NHEJ pathway (Cornet et al., 2018). HDR-based knockins rely on single-stranded oligodeoxynucleotide or plasmid as donor DNA for precise sequence integration, with plasmids used for the incorporation of longer sequences (Cornet et al., 2018). HDR methods have been used in the zebrafish for the precise placement of disease-associated point mutations (Armstrong et al., 2016; Zhang et al., 2017), stop codon cassette (Gagnon et al., 2014), LoxP sites (Hoshijima et al., 2016), and epitope-tagged or reporter proteins (Hoshijima et al., 2016; Eschstruth et al., 2020), albeit at typically much lower efficiency than INDELs (Cornet et al., 2018). Current methods toward improving HDR efficiency include pharmacologically or genetically enhancing HDR over NHEJ, cell cycle synchronization to HDR-active phases, donor template modification, and Cas9 engineering (Aksoy et al., 2019; Liu K. et al., 2019; Liu M. et al., 2019). In addition to HDR, homology-independent NHEJ knockin could be used for targeted plasmid integration *via* simultaneous cutting of the target DNA and the plasmid donor; this method offers a controlled approach for transgenesis (Auer et al., 2014; Kimura et al., 2014; Kesavan et al., 2017; Cornet et al., 2018). In the context of LSD modeling, while the lower efficiency of HDR knockins is a likely contributor toward the current scarcity of zebrafish knockin models relative to null mutants, the precision of the former renders it a promising tool for the modeling of well-defined LSD point mutations.

### Improving Model Generation Speed

Another limitation associated with the zebrafish system is the speed of model generation. Following rapid early development, zebrafish reaches sexual maturity in 10–12 weeks (Westerfield, 2007), and has a mean lifespan of 36–42 months depending on strain (Gerhard et al., 2002). While increased CRISPR-Cas9 efficiency and sequencing speed support high-throughput gene editing and phenotypic screening in F0 or F1 fish (Shah et al., 2015; Varshney et al., 2015), the creation of an isogenic mutant (F2 or F3) demands at least two rounds of propagation, or approximately 6 months given the 3 months typically required for each generation to reach reproductive age. Consequently, the maturation time of the zebrafish, as well as efforts required for individual genotyping, are comparable to those of some rodent systems. Notably, another teleost species, the African turquoise killifish, possesses a natural lifespan of 4–6 months and has gained significant traction as a vertebrate model in aging studies (Hu and Brunet, 2018). While development-specific bottlenecks could not be entirely circumvented in the zebrafish, recent technological innovations in larva genotyping provide solutions toward shortening the model generation process.

Genotyping in the zebrafish is routinely performed on adult fish *via* finclipping, in which a small portion of the tail fin is removed under anesthesia and digested to yield genomic DNA. Manual finclipping for a 96-well plate could typically be completed in 2–3 h. While adult finclipping is sufficient for line propagation and characterization of late-onset phenotypes, larva genotyping is required in instances where phenotypic characterization is performed on larvae from the incross of heterozygous parents. Unlike adult fish, larva finclipping could be challenging and time-intensive given the latter’s small size, and is less feasible for studies involving tail injury models. While genotyping could be performed on whole larvae post-experiment, this requires euthanization of the organism, and inclusion of larger larva numbers during experimentation as only a portion of the population is expected to carry the mutation of interest.

To address the aforementioned issues, several approaches have been developed that rely on genetic material from fin (Samuel et al., 2015), chorionic fluid (Samuel et al., 2015), or skin (Lambert et al., 2018; Zhang X. et al., 2019). Using replica molding, Samuel et al. (2015) designed two microfluidic devices that allow isolation of either chorionic fluid from individual embryo *via* microchannel-facilitated chorion rupture, or fin tissue from individual larvae *via* a multichannel system for larva positioning and fin removal, at a success rate of 78% for the former and 100% for the latter. More recently, the ZEG (Zebrafish Embryo Genotyper) system was developed that removes skin tissue from embryos or larvae *via* vibration over a rough glass surface at the rate of 24 fish per 10 min, with >90% success rate and no subsequent changes in larva behavior (Lambert et al., 2018). Another method toward skin cell isolation involves controlled enzyme digestion; by carefully optimizing proteinase K treatment parameters, Zhang X. et al. (2019) have demonstrated isolation of genetic material from larval skin tissue in a 96-well plate with >95% success rate and >90% viability. Taken together, these methods provide different options toward rapidly obtaining genotype information at the embryo or larval stage.

### Expanding Optical Clarity

Given the prominent involvement of neurodegeneration across LSDs, the ability to image deep into the brains of LSD models could broaden understanding of potential perturbations across brain cell populations. Optical clarity of zebrafish embryos and larvae, coupled with the availability of transgenic zebrafish displaying cell type-specific fluorescence, is well suited toward imaging studies of the brain and peripheral organs. Zebrafish possess several pigment cell types including the neural crest-derived melanophores, iridophores, and xanthophores (Antinucci and Hindges, 2016), and the optic neuroepithelium-derived pigment cells that form the retinal pigmented epithelium (Bharti et al., 2006). During normal development, zebrafish gradually lose optical transparency due to increased pigmentation, and becoming mostly opaque in the juvenile stage (Parichy et al., 2009).

Common approaches toward pigment reduction could largely be categorized as pharmacological or genetic (Antinucci and Hindges, 2016). Phenylthiourea is a widely used melanogenesis inhibitor that is typically added to the embryo medium at 12–24 hpf, but has been reported to alter gene expression, and cause morphological changes such as decreased eye size (Li et al., 2012). Many zebrafish pigment mutants have been identified, some of which also exhibit organogenesis defects that lead to early death, due to mutations that perturb neural crest development (Lister, 2002). The *casper* (*roy*; *nacre*) zebrafish (White et al., 2008), first developed in 2008 as a tool for improved tumor engraftment visualization, is a combinatorial pigmentation mutant derived from the melanocyte mutant *nacre* (*mitfa^–/–^*; Lister et al., 1999) and the iridophore mutant *roy orbison* (*mpv17^–/–^*; Ren et al., 2002; D’Agati et al., 2017). *casper* zebrafish remain devoid of melanocytes and iridophores through all stages of life (White et al., 2008). As the body of the *casper* fish is close to transparent, major peripheral organs can be visualized by stereomicroscopy, although the brain remains less visible due to the translucent skull (White et al., 2008). More recently, further gene editing based on the *casper* background resulted in *crystal* (*nacre; alb;* and *roy*), a *casper*-like fish that also lacks melanin in the retinal pigmented epithelium, thus facilitating optical investigations (Antinucci and Hindges, 2016).

While *crystal* and *casper* zebrafish have significantly improved visualization of the eyes and peripheral organs, the zebrafish skull adds opacity to the head region. Unlike the zebrafish, *Danionella translucida*, a species of freshwater Danionin fish from Myanmar, remains optically transparent across all stages of life (Schulze et al., 2018). As the skull of *Danionella translucida* only surrounds the brain laterally and ventrally, imaging from the dorsal side yields an unblocked view of the brain (Schulze et al., 2018). Recently, Schulze et al. (2018) demonstrated the presence of complex behaviors in *Danionella translucida*, and established Tol2 transgenesis and CRISPR-Cas9 protocols for this fish model, thus paving the way for future disease modeling.

### Chemical Screening With Zebrafish LSD Models

Given the current lack of treatment options for the majority of LSDs, there exists an urgent and unmet need for drug discovery. As each zebrafish pair can yield over a hundred embryos weekly, thousands of embryos could be obtained at once for chemical screening. Many of the existing zebrafish LSD models demonstrate screenable phenotypes such as craniofacial defects, microphthalmia, and altered behavior (Supplementary Table S1). As different neuronal features could be labeled *via* transgenic lines (Marquart et al., 2015; Förster et al., 2017; Tabor et al., 2019) or antibody staining (Brösamle and Halpern, 2009; Staudt et al., 2015), imaging-based screens could be conducted on zebrafish LSD models to identify modulators of neuronal processes such as axon tracking, myelination, microglia activation, and calcium signaling (Early et al., 2018). Given it is possible to obtain detailed metabolomic information from a relatively small number of larvae (Fraher et al., 2016), high-throughput LC–MS-based methods could provide another avenue toward the identification of compounds that directly inhibit substrate accumulation.

Since the first implementation of zebrafish larvae in a 96-well screening format in 2000 (Peterson et al., 2000), over one hundred zebrafish chemical screens have been conducted (Zhang and Peterson, 2020). As screen design increases in complexity, some of the associated challenges include larva orientation and screening in older animals. Larval zebrafish typically rest laterally prior to swim bladder inflation around 5 dpf (Kimmel et al., 1995), following which larvae adopt a dorsal-ventral orientation and demonstrate a wider range of locomotive behaviors. Given the frequent use of transgenic zebrafish in imaging-based screens, it can be crucial that larvae remain stationary and consistently oriented across all wells. Technologies for optimizing larval positioning include the SideView plate that facilitates lateral imaging (Rovira et al., 2011), 3D printing of customizable molds for agarose-filled multiwells (Wittbrodt et al., 2014), microfluidic device for head immobilization that leaves the tail region free for behavioral monitoring (Khalili et al., 2019), and the VAST BioImager Platform (Union Biometrica) that positions individual larva in a capillary for imaging (Pardo-Martin et al., 2010; Chang et al., 2012; Pardo-Martin et al., 2013). Coupling of the VAST platform with confocal microscopy enabled high throughput imaging of myelinating oligodendrocytes in transgenic zebrafish larvae, which was expanded into a chemical screen that identified several enhancers of myelinating oligodendrocyte formation (Early et al., 2018). As demyelination is a common feature of many LSDs, the combination of zebrafish LSD models with the VAST screening strategy could facilitate identification of small molecules with the potential to alleviate myelin loss.

Compared to screening of embryos or larvae, screening of juvenile or adult zebrafish presents new challenges given the latter’s increased size and lack of optical transparency. While less common, chemical screens have been conducted in adult fish with focuses on regeneration (Oppedal and Goldsmith, 2010), behavior (Maximino et al., 2014), and transplantation biology (Li et al., 2015). In addition, recent implementation of adult zebrafish in complex behavioral assays such as social recognition memory (Madeira and Oliveira, 2017) and opioid self-administration (Bossé and Peterson, 2017) supports a corresponding expansion in screening platforms to explore pathways relevant to all stages of life. Importantly, fish-based screens need not be restricted to one species. The previously discussed *Danionella translucida*, which lack dorsal skull structure and remain optically clear through all stages of life, are significantly smaller than zebrafish and similarly amenable to mutagenesis (Schulze et al., 2018). As a complement to existing zebrafish LSD models, adaption of *Danionella translucida* toward chemical screening could provide a novel avenue for the discovery of neuro-modulatory compounds in both larval and later developmental stages.

## Conclusion

In this review, we have highlighted the zebrafish as a model organism for LSDs. A survey of the existing literature identified 60 zebrafish LSD models with published phenotypes, many of which recapitulated known disease pathologies. 55% of the models were transient knockdowns, while the rest were stable mutants. Notably, the rise in TALEN and CRISPR-Cas9 models over the past 3 years is a reflection of the recent successful implementation of these gene editing methods in the zebrafish. Disruptions in major signaling pathways were observed across several zebrafish models, and pharmacological modulations of some of these pathways resulted in rescue of LSD pathology. Taken together, advantages of the zebrafish system include conservation of major organs and metabolic pathways, ease of genetic manipulation, optical transparency during early stages, and amenability toward high-throughput screening. Conversely, the zebrafish system also presents limitations in the speed of model generation, genome duplication, added opacity across later life stages, and screen-related challenges such as orientation and size restrictions. While some of these drawbacks are not circumventable due to organism-specific biology, the emergence of additional teleost models such as the short-lived killifish species and the transparent *Danionella translucida* open up additional avenues for exploring disease biology. As zebrafish disease modeling looks toward the future, improvements upon existing gene editing methods will likely translate to an increase in both the quantity and precision of zebrafish LSD models, which will allow the investigation of additional disease-associated pathways. Correspondingly, technological innovations such as high-throughput genotyping and automated larva positioning could curtail existing bottlenecks, and accelerate the transition from model characterization to drug discovery.

## Author Contributions

TZ performed the survey of the literature and wrote the manuscript. RP provided supervision and recommendations on LSD-related publications. TZ and RP edited the manuscript.

## Conflict of Interest

The authors declare that the research was conducted in the absence of any commercial or financial relationships that could be construed as a potential conflict of interest.

## Footnotes

1 https://zfin.org
